# Telehealth Delivery of the Homeostasis–Enrichment–Plasticity Approach for Premature Infants With Developmental Risks: Exploratory Feasibility Study

**DOI:** 10.2196/86883

**Published:** 2026-04-07

**Authors:** Aymen Balikci, Gamze Cagla Sirma, Izgi-Miray Demirbag, Teresa A May-Benson, Hatice Gulhan Sozen, Ayse Firdevs Aracikul Balikci, Gul Ilbay

**Affiliations:** 1 Sense On Ltd Istanbul Turkey; 2 Department of Occupational Therapy Faculty of Health Sciences Fenerbahçe University Istanbul, Istanbul Turkey; 3 TMB Education Norristown, PA United States; 4 Department of Child Health and Diseases Faculty of Medicine Bahcesehir University Istanbul Turkey; 5 Department of Special Education Faculty of Education Istanbul Medipol University Istanbul Turkey; 6 Department of Physiology Faculty of Medicine Kocaeli University Kocaeli Turkey

**Keywords:** early intervention, environmental enrichment, premature infants, telehealth, adherence, retention

## Abstract

**Background:**

Preterm delivery is an increasing worldwide health concern linked to increased neurodevelopmental risks. Early intervention is crucial for harnessing neuroplasticity to enhance developmental and functional performance outcomes; however, access to early intervention is frequently hindered by logistical, financial, and labor constraints. The Homeostasis–Enrichment–Plasticity (HEP) Approach is a family-centered early intervention model based on enriched environments, designed to improve infants’ sensory-motor, cognitive, and socio-emotional development.

**Objective:**

This study aimed to assess the feasibility, safety, acceptability, and outcomes sensitivity to change of implementing the HEP Approach through telehealth for premature infants at developmental risk.

**Methods:**

A pre-post exploratory feasibility study was performed, including 16 preterm infants (aged 4-12 months corrected age), of whom 14 completed the study. The 12-week intervention included weekly remote sessions focused on environmental enrichment, active exploration, and parental guidance. The feasibility and acceptability were evaluated using a 24-item questionnaire. Developmental outcomes were assessed with the Young Children’s Participation and Environment Measure, Ages and Stages Questionnaire (ASQ), Alberta Infant Motor Scale, Infant Motor Profile, and Depression Anxiety Stress Scales.

**Results:**

High adherence (14/14, 100%) and retention (14/16, 87.5%) rates demonstrated robust feasibility. Parents indicated 86%-100% agreement across all feasible criteria, affirming safety, satisfaction, and acceptability. No adverse incidents were reported. Changes were identified in participation (Young Children’s Participation and Environment Measure), motor development (Alberta Infant Motor Scale, Infant Motor Profile, and ASQ), communication and social-emotional domains (ASQ), and caregiver well-being (Depression Anxiety Stress Scales) (*P*<.05).

**Conclusions:**

The telehealth implementation of the HEP Approach demonstrated feasibility, safety, and strong acceptance among families, along with quantifiable developmental and psychosocial changes. These initial findings endorse the model’s viability as an accessible, family-oriented telehealth framework for infants born preterm. Future randomized controlled and longitudinal studies are necessary to validate intervention efficacy and scalability.

## Introduction

Approximately 1 in every 10 children worldwide (9.9% of all births) is born preterm (<37 weeks of gestation) [1]. Preterm delivery is associated with substantial long-term health consequences, spanning multiple domains such as respiratory and cardiovascular health as well as neurodevelopmental concerns. Within the neurodevelopmental domain, impairments may range from severe conditions, such as cerebral palsy—particularly among those born at the lowest gestational ages—to less severe but still clinically significant outcomes [1-3]. Recent evidence indicates that even births occurring just a few weeks early increase the risk of adverse developmental outcomes [1,4,5]. This is particularly relevant as approximately 85% of preterm births occur between 32 and 37 weeks of gestation, meaning that a large proportion of preterm infants are at risk, challenging the earlier assumption that only extremely preterm infants face vulnerability [1,4,6]. Moreover, numerous studies have demonstrated that, compared with their full-term-born peers, preterm infants are at heightened risk for difficulties across multiple developmental domains, including cognitive [7-9], motor [7,10-12], sensory [11-13], language [11,14,15], and social-emotional functioning [5,15,16].

Overall, these data emphasize the importance of early developmental support for infants born preterm [17,18]. Given the significant neuroplasticity in early life [19,20], early intervention (EI) can influence multiple domains related to prematurity [17]. Evidence indicates that structured services during infancy improve cognitive, motor, sensory, language, and social-emotional outcomes, while also fostering long-term neurodevelopmental pathways and functional engagement [12,21-24]. Consequently, EI may influence neurodevelopmental trajectories, reducing risks associated with prematurity and enhancing the quality of life for infants and their families [12,17,18,24-26].

Although EI provides substantial benefits for the development of preterm infants, many families face persistent challenges in accessing these services. The most prominent barriers include shortages of qualified professionals, transportation difficulties, and the difficulty of organizing time to attend services—factors that are especially evident for families living in rural or underserved areas. While systemic issues such as delayed referrals or fragmented communication may also play a role, it is these fundamental constraints of workforce availability, accessibility, and time that most directly limit timely participation in EI and reduce its potential impact during critical developmental windows [27-30].

With advances in digital technologies, telehealth has become a promising alternative for addressing barriers to EI in preterm infants [31,32]. Studies during the COVID-19 pandemic showed that telemedicine maintained developmental follow-up and medical care when in-person visits were restricted, mitigating workforce and logistical challenges [33]. Telehealth services are valuable for infants discharged from the neonatal intensive care unit, as they decrease needless hospital visits and limit exposure to transmissible infections [31,32,34]. Furthermore, studies demonstrate that telehealth can be as efficacious as in-person care for many health conditions and interventions [31,35,36] and may offer a cost-effective health care delivery paradigm [31,32,37,38].

Building on the growing evidence for telehealth as a viable and cost-effective model of early intervention, the Homeostasis–Enrichment–Plasticity (HEP) Approach [39] represents a structured framework that can be adapted to remote delivery while preserving its core principles of parent coaching and enriched developmental support. The HEP Approach, developed by Balikci [39], is a comprehensive EI model designed to promote sensory-motor, cognitive, and social-emotional development in infants. The HEP Approach incorporates 10 fundamental principles—homeostasis, safety, multisensory experiences, novelty, sustained engagement, and active exploration—based on environmental enrichment paradigms, through a clinic-based program that includes parent coaching and concurrent home implementation [24,39,40]. The approach provides infants with enhanced sensory and motor experiences in the clinic, while parents obtain systematic coaching that is later integrated into everyday routines and natural settings, with telehealth follow-up reinforcing these procedures at home. Rooted in frameworks including Ecological Perception Theory, Perception–Action Theory, Sensory Integration, the Person–Environment–Occupation (PEO) Model, and Dynamic Systems Theory, the HEP Approach equips caregivers with adaptable, goal-focused strategies that enhance co-regulation, sensory-motor learning, and adaptive participation [24,39,40].

While previous research has demonstrated the feasibility and effectiveness of the HEP Approach in the traditional clinic-based/home-monitored format [24,40], its applicability through telehealth has not yet been systematically examined. This study represents the first attempt to evaluate the feasibility of delivering the HEP Approach via telehealth.

The aim of this study is to assess the feasibility of implementing the HEP Approach in a telehealth format and to determine whether this model provides an accessible, acceptable, and practical option for supporting the developmental outcomes of premature infants and their families.

## Methods

### Study Design

This is a pre-post feasibility experimental study. The study was conducted in Istanbul, Türkiye, with interventions delivered remotely via telehealth between January 2025 and July 2025.

### Ethical Considerations

The study was conducted in accordance with the Declaration of Helsinki and approved by the Clinical Research Ethics Committee of Istanbul Medipol University (E-10840098-202.3.02-7368). All caregivers provided written informed consent before participation and were informed of their right to withdraw from the study at any time. Participant privacy and confidentiality were strictly maintained, and all data were anonymized prior to analysis. No identifiable personal information was included in the manuscript. No financial or material compensation was provided to the participants.

### Study Procedures

The study comprised 11 structured phases that guided the implementation of the HEP Approach via telehealth [40]. Phase 1 covered participant recruitment via physician referral and digital record review, while Phase 2 introduced families to the HEP philosophy and telehealth process. Phase 3 of the study comprised baseline evaluations completed exclusively remotely via Zoom. The evaluation process comprised caregiver interviews, unstructured observations, and structured observations tailored to the requirements of standardized assessment instruments. All evaluations, including the implementation of standardized assessments, were conducted remotely via videoconferencing (Zoom). In Phases 4-5, assessment findings were collaboratively interpreted with families, and hypotheses regarding sensory-motor, emotional, social, and environmental factors were formulated and discussed. Phase 6 focused on interactive goal setting, followed in Phases 7-8 by individualized intervention strategies delivered through weekly telehealth coaching, demonstrations, and integration into daily family routines, supported by video sharing via WhatsApp. Phase 9 emphasized adaptation and generalization of strategies, while Phase 10 ensured continuous monitoring with therapist feedback and reflective questioning to enhance family problem-solving. Finally, Phase 11 included postintervention assessments, feasibility questionnaires, and feedback interviews conducted via videoconferencing. A comprehensive description of each phase is provided in Table S1 in Multimedia Appendix 1.

Fidelity to the intervention was maintained through multiple strategies. Treating therapists completed 120 hours of theoretical and practical training, including supervision, provided by the first author, who developed the model. In addition, fidelity was reinforced through regular mentoring and the first author’s systematic review of recorded telehealth sessions. All procedures were implemented in accordance with the HEP Approach Implementation Manual [22], and session recordings were evaluated using the fidelity checklists included in the manual to ensure high implementation reliability.

### Participants and Recruitment

Convenience sampling, a nonprobability method, was used [40]. Neonatologists from 3 hospitals in Istanbul referred premature infants who met the designated inclusion criteria. The inclusion criteria were (1) corrected age ranging from 4 to 12 months, (2) birth at ≤36 weeks and 6 days of gestation, (3) absence of systemic diseases or congenital defects, and (4) familial consent to participate consistently in the study process. The exclusion criteria included the following conditions: (1) substantial impairment of vision or hearing; (2) history of febrile seizures; (3) medical conditions that impede active participation in the study, such as dependence on oxygen; and (4) prior participation in other experimental rehabilitation studies. Following assessment of the qualifying criteria, informed consent was secured.

**Figure 1:**
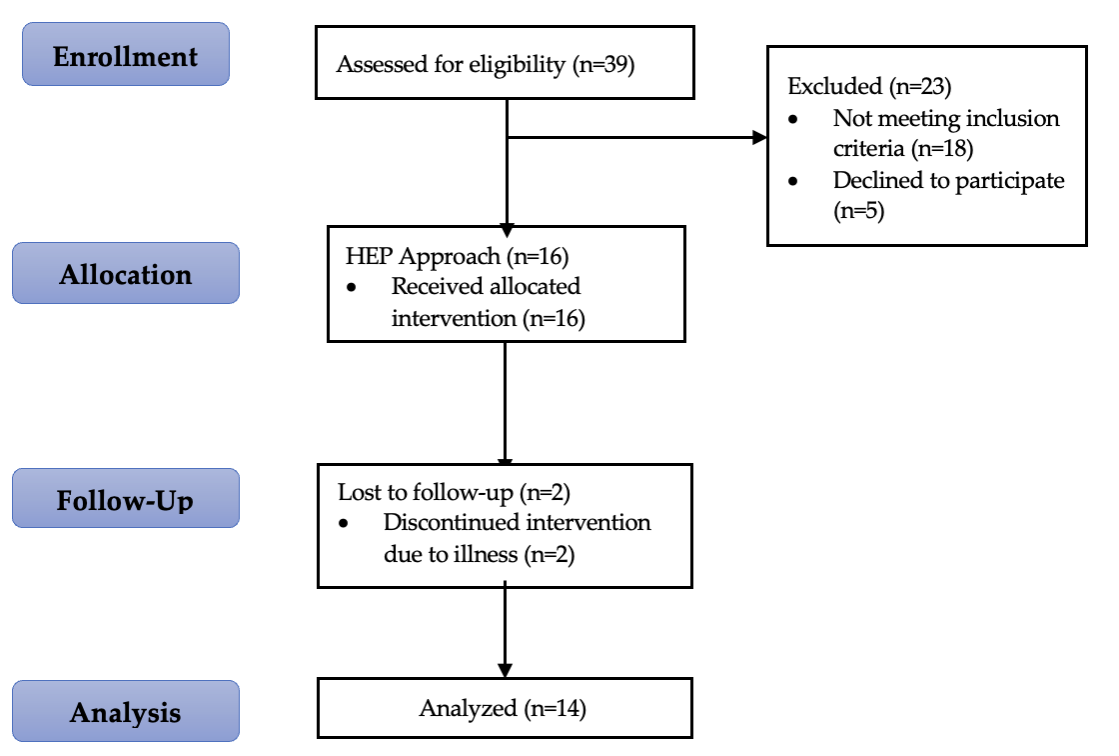
Flowchart of participant and outcome assessment through the trial. HEP: Homeostasis–Enrichment–Plasticity.

A total of 39 preterm infants were assessed for eligibility. Eighteen did not meet the inclusion criteria, and five families declined to participate, resulting in 16 infants enrolled in the HEP telehealth intervention. All 16 received the intervention. During follow-up, 2 infants discontinued participation due to illness, leaving 14 who completed the program and were included in the final analysis. All pre- and postintervention evaluations were conducted by a physical therapist with over 5 years of pediatric experience and an advanced Master of Science degree. This therapist had received formal training in the administration and scoring of outcome measures as part of their graduate program and was blinded to the intervention. The HEP Approach itself was delivered by 2 physiotherapists, each with more than five years of pediatric clinical experience and advanced Master’s-level training. The participant flow is illustrated in Figure 1.

### Feasibility Measure

Feasibility and acceptability of the telehealth-based HEP Approach were evaluated using a 24-item Likert-type questionnaire, adapted from the feasibility study by Balikci et al [40]. The survey was structured to assess four domains: feasibility (items 1-7), acceptability (items 8-12), satisfaction (items 13-19), and safety (items 20-24). Each item was rated on a five-point scale ranging from strongly agree to strongly disagree. For analytic purposes, responses of “Strongly Agree” and “Agree” were categorized as agreement, whereas all other responses were classified as disagreement. Alongside the structured items, 3 open-ended questions were incorporated at the conclusion of the form, inviting parents to provide their responses. The qualitative data were subsequently examined and thematically grouped according to the 3 questions. Feedback on the intervention’s feasibility, safety, and perceived effectiveness was also collected from both therapists through postintervention reflections and monitoring logs maintained throughout the study period.

### Outcome Measures

Caregivers first completed a demographic questionnaire capturing infant and maternal health, gestational and postnatal history, corrected age at enrollment, gender, birth weight, gestational age, maternal age, family income, and caregiver educational level.

Five standardized outcome measures were used to evaluate developmental progress across multiple domains. The Turkish translated version of the Young Children’s Participation and Environment Measure (YC-PEM) was used to assess participation frequency, involvement, desire for change, and environmental support in both home and community contexts [41-43]. Broader developmental domains, including social-emotional, communication, gross motor, fine motor, problem-solving, and personal-social skills, were measured with the Turkish translated version of the Ages and Stages Questionnaire (ASQ) [44]. Motor development was further assessed using the Alberta Infant Motor Scale (AIMS) [45-49] and the Infant Motor Profile (IMP) [50-52], which captured dimensions such as variation, adaptability, symmetry, fluency, and performance. Finally, parental mental health and well-being were evaluated with the Depression Anxiety Stress Scales (DASS-21) [53]. The selection of outcome measures was guided by the study’s feasibility objectives rather than by an intention to establish intervention efficacy. AIMS and IMP were selected as standardized, performance-based tools reported in remote or telehealth assessment contexts and among the limited motor assessments adaptable to video-based administration. In this study, these instruments were used to evaluate (1) whether they could be feasibly administered in a telehealth format within our sample and (2) whether they were capable of detecting measurable change over a 12-week period, regardless of whether such change reflected intervention-related or developmental factors.

Caregiver-reported measures (YC-PEM, ASQ, and DASS-21) were included to capture domains not directly assessable via remote standardized performance testing and to examine the feasibility of administering structured family-report instruments within a telehealth early intervention framework.

### The Young Children’s Participation and Environment Measure-Turkish Version

The Turkish translation of the YC-PEM was used in this study. The YC-PEM is a caregiver-completed measure designed to assess children’s participation up to 5 years of age across home, daycare/preschool, and community settings. It evaluates four domains: frequency of participation, level of involvement, parental desire for change, and environmental supports and barriers. Previous studies demonstrated good internal consistency (α=0.67-0.96), fair-to-good test-retest reliability (ICC=0.59-0.94) in home and community contexts, and adequate construct validity in differentiating participation across age and disability groups [41,42]. The Turkish translation and cross-cultural adaptation of the YC-PEM were conducted following standardized procedures, and subsequent psychometric evaluation in a large sample of 367 children, both with and without disabilities, demonstrated good reliability (Cronbach α=0.68-0.94; ICC=0.69-0.89) together with evidence of discriminant and concurrent validity, thereby supporting the YC-PEM-T as a reliable and culturally appropriate instrument for assessing children’s participation and environmental influences in Türkiye [43]. The YC-PEM was provided to families in electronic (PDF) format. Caregivers independently completed the measure at their convenience and returned the completed forms electronically.

### The Ages and Stages Questionnaire-Turkish Version

The Turkish version of the Ages and Stages Questionnaire –Turkish Version (ASQ-TR) was used. The ASQ is a parent-completed developmental screening instrument designed for children aged 4 to 60 months (extended up to 72 months in some adaptations). Each form consists of age-specific items that evaluate five developmental domains: communication, gross motor, fine motor, problem-solving, and personal-social skills. Parents complete the questionnaires in approximately 10-15 minutes, based on their observations and knowledge of their children’s performance, and respond to items with options such as “yes,” “sometimes,” or “not yet.” Responses are scored to determine whether the child’s development is on schedule, requires monitoring, or needs referral for further evaluation.

The original ASQ demonstrated good psychometric qualities, with reported sensitivity values typically ranging from 75% to 90% and specificity from 77% to 88%, alongside strong reliability indices and evidence of construct validity against standardized developmental assessments. The Turkish version (ASQ-TR), evaluated in a large sample of 833 children aged 3-72 months, demonstrated satisfactory reliability and validity. Concurrent validity analyses showed overall agreement rates of 70.95% when one domain was below the cutoff and 88.24% when 2 domains were below the cutoff, supporting the 2-domain criterion as a more accurate classification approach. Internal consistency coefficients (Cronbach α) ranged from 0.38 to 0.95 across domains and age forms, generally increasing with age. Domain-total correlations (*r*=0.54-0.96) and interdomain correlations (*r*=0.65-0.79) were all statistically significant. Test-retest reliability over a 2-week interval indicated high agreement across domains (ranging from 0.82 to 0.97), and interrater reliability between mothers and teachers had agreement levels between 73.68% and 87.18%, depending on the criterion used.

The use of a 2-domain cutoff criterion considerably improved diagnostic accuracy in this version of the measure, increasing specificity to 85% and positive predictive value to 75%, while maintaining high sensitivity (94%). These findings support the ASQ-TR as a reliable and culturally appropriate developmental screening tool for use in Türkiye. Families received the ASQ-TR in PDF format; caregivers completed the measure at their convenience and submitted the forms electronically.

### The Alberta Infant Motor Scale

The AIMS is a standardized observational tool developed to identify motor delays and describe infants’ gross motor performance. It evaluates spontaneous movements, posture, weight shifts, and antigravity control over a 20-30 minute assessment, often supported by age-appropriate toys to encourage activity. The scale includes 58 items across four positions (21 prone, 9 supine, 12 sitting, 16 standing), scored dichotomously and summarized on age-specific percentile charts ranging from the 5th to the 90th percentile. Based on these scores, infants’ motor performance can be classified as delayed, suspect, or normal [45,46]. Due to its low cost, ease of administration, reproducibility, and minimal handling of the infant, AIMS is widely recognized as a practical measure in both clinical and community health contexts, and has been validated as reliable for high-risk populations The AIMS demonstrated strong concurrent validity with the Bayley Scales of Infant Development (*r*=0.95; *P*<.01) and high interrater reliability across all tested ages (intraclass correlation coefficients ranging from 0.76 to 0.99), supporting it as a valid and reliable tool for assessing motor development in high-risk infants [47]. More recently, studies have demonstrated that the AIMS can be successfully applied in telehealth settings, with strong agreement between remote and face-to-face assessments for neurodevelopmentally high-risk infants [48,49]. In this study, the AIMS was administered remotely via videoconferencing (Zoom). The evaluating specialist provided real-time verbal guidance to caregivers on how to position their child for each assessment item and scored the child’s performance based on live observations. All sessions were video-recorded, enabling the evaluator to revisit the recordings when needed to verify or adjust scores. This administration procedure aligned with protocols reported in prior research [48,49].

### The Infant Motor Profile

The IMP is a video-based observational tool designed to evaluate motor behavior in infants aged 3 to 18 months, or until a few months after independent walking is achieved. It consists of 80 items across five domains—movement variation, adaptability, symmetry, fluency, and performance—capturing both quantitative and qualitative aspects of motor development. Grounded in the Neuronal Group Selection Theory, the IMP emphasizes the importance of motor variability and adaptive strategy selection as indicators of central nervous system integrity [50].

The IMP has demonstrated strong psychometric properties, including acceptable interrater reliability in high-risk infants, with Spearman correlation coefficients ranging from 0.47 to 0.94 across domains; reliability was highest for the performance domain (*r*=0.90-0.93) and lowest for symmetry (*r*=0.47-0.74), supporting its use as a reliable tool for assessing early motor development and identifying physiotherapy needs in the Turkish sample [51]. Moreover, the IMP has shown excellent concurrent validity with the AIMS (ρ=0.76; *P*<.001), a significant association with the General Movement Assessment, and high diagnostic accuracy in predicting neurodevelopmental disorders at 5 months (AUC=0.92), with strong sensitivity (93%) and specificity (81%), confirming its validity and clinical utility for evaluating early motor behavior and reflecting the severity of brain injury in at-risk infants. [52]. Additionally, studies indicate that the IMP is responsive to change, making it a valuable outcome measure in early intervention research [52]. Collectively, these findings support the IMP as a reliable and valid tool for assessing motor development and detecting early deviations in infants at risk of neurodevelopmental disorders. The IMP was administered by the examiner, who provided structured instructions for various positions and object manipulations during the initial session; the examiner recorded the session and took detailed notes while guiding the caregiver through the tasks, and later used the video recording to score and analyze the child’s performance.

### Depression Anxiety Stress Scales-21-Turkish Version

The DASS-21 is a self-report instrument designed to assess levels of depression, anxiety, and stress [53]. The Turkish cultural adaptation, validity, and reliability study of the DASS-21 was conducted by Yılmaz et al [54], who refined the existing Turkish translation of the scale through a comprehensive linguistic and cultural adaptation process and subsequently confirmed its psychometric soundness. Proven to be a valid and reliable tool for screening depressive and anxiety disorders, the DASS-21 effectively evaluates the severity of these 3 psychological conditions through 21 items. The scale consists of 3 subscales: depression (7 items), anxiety (7 items), and stress (7 items). Each item is rated on a 4-point Likert scale ranging from 0 (never) to 3 (always). Lower scores on the subscales indicate better psychological well-being [54]. The electronic (PDF) version of the DASS-21 in Turkish was given to families. At their convenience, caregivers completed the measure themselves and submitted the completed forms via WhatsApp.

### Intervention

The HEP Approach, developed by Balıkcı [39], is a systematic early intervention model grounded in EE paradigms, principles of neuroplasticity, and ecological-dynamic theories of human development. Drawing on experimental EE research and mechanisms of brain plasticity, the model translates these principles into clinical practice through an integrated theoretical framework that combines Ecological Theory, Dynamic Systems Theory, Gibson’s perception-action perspective, the Theory of Neuronal Group Selection, sensory integration theory, and the PEO model. Within this framework, development is conceptualized as emerging from the continuous interaction among the child, task demands, environmental affordances, and temporal processes [39,40].

Ecological and PEO perspectives inform the alignment of environmental supports and task structures with the infant’s regulatory and developmental capacities, particularly within the zone of proximal development. Dynamic Systems Theory underpins the emphasis on non-linear change and self-organization, operationalized clinically through graded challenge, structured variation, and strategic manipulation of environmental and task constraints to facilitate spontaneous motor and behavioral reorganization. Gibson’s perception-action framework guides the design of environments that invite active exploration, ensuring that sensory information is directly coupled with meaningful action. The Theory of Neuronal Group Selection and neuroplasticity principles support the systematic use of novelty, repetition within variation, and sustained enriched exposure to strengthen adaptive neural networks through experience-dependent plasticity.

Central to the HEP model is physiological homeostasis, conceptualized as a foundational prerequisite for exploration, engagement, and learning. Regulation of sleep, arousal, stress modulation, and coregulation processes is addressed prior to and throughout intervention to optimize the infant’s readiness for adaptive change. Enriched environment principles are operationalized through 10 structured intervention components: physiological homeostasis, safety, provision of multisensory experiences, spatial adaptation, environmental and object novelty, just-right challenge, enjoyment, social opportunities, continuity, and active engagement and exploration. These components guide assessment, hypothesis generation, and intervention planning by systematically aligning environmental affordances, task demands, and individual sensory-motor and regulatory capacities.

In practice, the HEP Approach integrates structured therapist guidance with caregiver coaching to embed enriched, developmentally attuned experiences into daily routines and natural contexts. More detailed information regarding the theoretical foundations and conceptual framework of the HEP model is available in previously published studies [22,24,39,40].

The approach consists of 11 structured phases, beginning with referral and family introduction, followed by comprehensive assessment, identification of child and family strengths and challenges, hypothesis formulation, collaborative goal setting, selection of outcome measures, intervention planning, implementation, monitoring, and evaluation [40]. In this study, all phases of the HEP Approach were adapted for telehealth delivery and implemented over 12 one-hour weekly sessions across 3 months. Families, guided through Zoom, arranged their home environments according to their own resources, preferences, and the child’s developmental needs, transforming everyday spaces into therapeutic contexts. Assessments and observations were conducted via structured caregiver interviews, live video interaction, and parent-recorded video clips, while therapists engaged parents in reflective discussions to interpret child behaviors and environmental affordances. Intervention planning and delivery progressed systematically: early sessions emphasized supporting homeostasis and self-regulation; subsequent sessions focused on reorganizing physical and social environments to foster active exploration; later sessions encouraged the diversification and generalization of newly acquired skills across settings and materials; and final sessions concentrated on empowering parents to independently adapt environments and sustain enriched opportunities.

**Figure 2:**
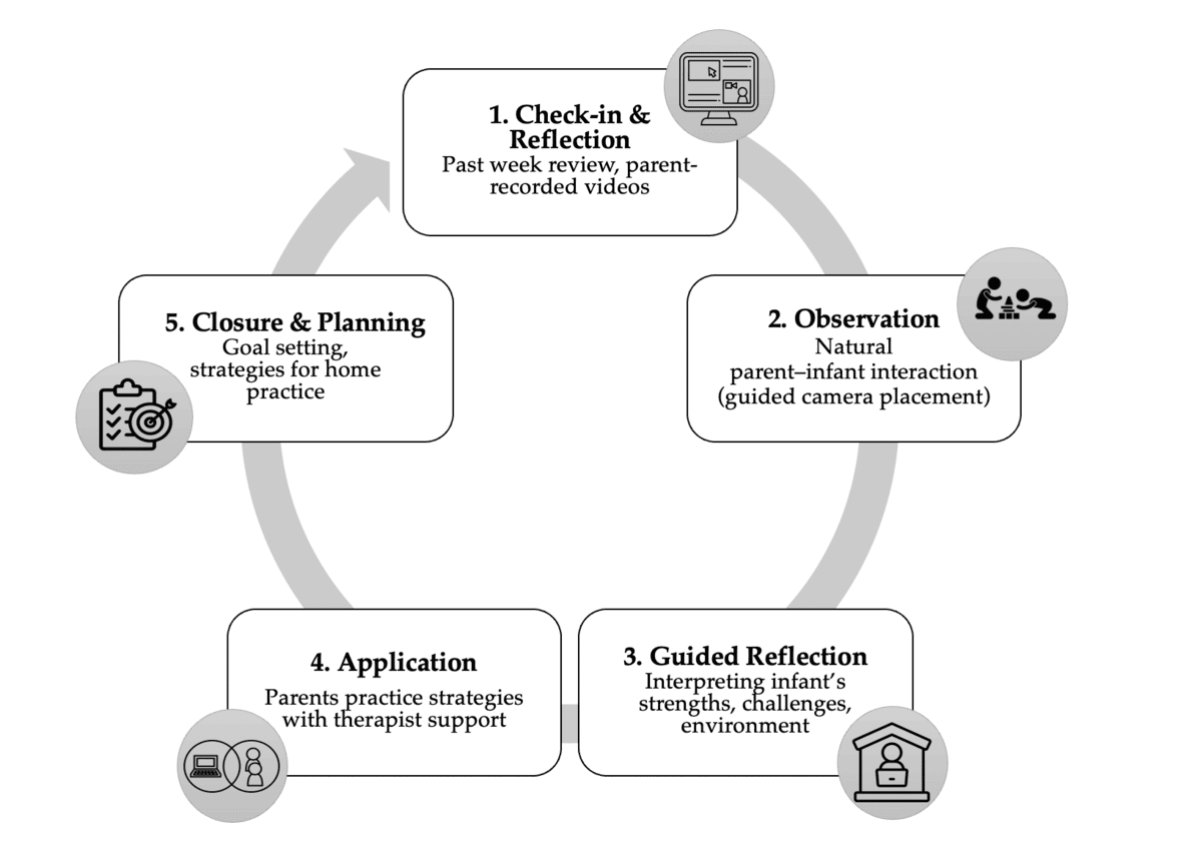
Therapeutic process cycle in telehealth sessions.

Each telehealth session also followed a five-step therapeutic sequence to ensure fidelity and caregiver engagement: (1) a check-in and reflection phase, where parents and therapists discussed the past week and reviewed parent-recorded videos; (2) an observation of natural parent-infant interaction, with therapists guiding camera placement to capture spontaneous exploration; (3) a guided reflective discussion, in which therapists and caregivers collaboratively interpreted the infant’s strengths, challenges, and responses to environmental affordances; (4) a real-time application phase, where parents directly practiced strategies with their infant during the session under therapist support; and (5) a closure and collaborative planning phase, setting goals and strategies for home practice until the next meeting (Figure 2).

Grounded in 10 core principles—including maintenance of homeostasis, sensory experiences, safety, novelty, spatial adaptation, just-right challenge, enjoyment, social opportunities, continuous engagement, and active exploration—the intervention sought not only to promote self-regulation, sensory processing, motor and functional skills in children, but also to enhance parenting self-efficacy, thereby enabling families to provide ongoing enriched environments across contexts [24,39,40]. Fidelity to the approach was assured through adherence to the HEP manual [22].

### Statistical Analysis

Study data were analyzed using IBM SPSS Statistics for Windows (version 22.0; SPSS Inc). Descriptive and demographic variables were summarized with frequency and percentage analyses. Outcome measure scores were examined using means and SDs. Normality of the research variables was assessed through skewness and kurtosis, with values between −1.5 and +1.5 for skewness and between −2.0 and +2.0 for kurtosis considered indicative of a normal distribution [55,56]. All variables met these criteria, confirming normal distribution. Accordingly, parametric statistical methods were applied. Two-sided paired *t* tests were conducted to compare repeated measures within dependent groups, and a *P* value of <0.05 was accepted as statistically significant. Given the exploratory feasibility design of the study, statistical analyses were conducted to examine whether measurable pre-post differences could be detected and to explore the sensitivity of the selected instruments to change within a telehealth context. No formal correction for multiple comparisons was applied, as the analyses were descriptive and hypothesis-generating rather than confirmatory.

## Results

### Demographic Characteristics of the Participants

The study included 14 infants, of whom 6 (42.9%) were female, and 8 (57.1%) were male. The mean maternal age at the time of birth was 33.42 (SD 3.69, range 25-39) years, while the mean paternal age was 34.85 (SD 4.46, range 27*-*44) years. Mothers predominantly held a bachelor’s degree (11/14, 78.6%), with a small proportion having completed high school (2/14, 14.3%) or postgraduate education (1/14, 7.1%). Similarly, most fathers had attained a bachelor’s degree (12/14, 85.7%), while 7.1% (n=1) had completed high school and 7.1% (n=1) held postgraduate qualifications. The mean gestational age at birth was 32.14 (SD 3.03, range 26*-*36) weeks, and the mean birth weight was 1951.57 (SD 748.08, range 855*-*3400) grams. At baseline, the corrected mean infant age was 8.03 (SD 1.53, range 4.5*-*10.5) months. Detailed demographic characteristics of the participants are presented in Table 1.

**Table 1:** Participant demographic information (N=14).

Characteristic		Values
**Sex, n (%)**		
	Female	6 (42.9)
	Male	8 (57.1)
**Mother’s educational level, n (%)**		
	High school	2 (14.3)
	Bachelor’s degree	11 (78.6)
	MSc or PhD	1 (7.1)
**Father’s educational level, n (%)**		
	Secondary school	0 (0)
	High school	1 (7.1)
	Bachelor’s degree	12 (85.7)
	MSc or PhD	1 (7.1)
**Maternal age at infant’s birth (years)**		
	Mean (SD)	33.42 (3.69)
	Min-Max	25-39
**Father’s age at infant’s birth (years)**		
	Mean (SD)	34.85 (4.46)
	Min-Max	27-44
**Birth gestational age (week)**		
	Mean (SD)	32.14 (3.03)
	Min-Max	26-36
**Corrected age at baseline (months)**		
	Mean (SD)	8.03 (1.53)
	Min-Max	4.5-10.5
**Birth weight (grams)**		
	Mean (SD)	1951.57 (748.08)
	Min-Max	855-3400

### Feasibility, Safety, Acceptability, and Parental Satisfaction in Telehealth

The trial enrolled 16 infants; however, 2 were withdrawn due to medical complications. One infant developed recurrent respiratory distress, and the other experienced feeding difficulties requiring hospitalization. As a result, both were unable to attend four consecutive telehealth sessions and were excluded from the study. Consequently, the intervention was completed with 14 infants who participated in all sessions and constituted the final sample for analysis. Over the 12-week period, all remaining caregivers and their infants attended the weekly sessions regularly and completed all 12 sessions without any disruptions.

The feasibility survey found highly positive results across all domains—feasibility, safety, acceptability, and parental satisfaction—regarding the implementation of the HEP Approach via telehealth. Overall, 86%*-*100% (n=12-14) of participants selected “Agree” or “Strongly Agree” across all survey items, indicating strong endorsement of the program. In terms of feasibility, 86% (n=12) of families agreed or strongly agreed that the HEP Approach was clear and understandable, and that the recommended activities and environmental arrangements were easy to apply within their daily routines. Additionally, 93% (n=13) found participation in remote sessions convenient, and the process of regularly sharing home activity videos with the clinician practical and manageable. The remaining responses represented participants who chose neutral options, reflecting uncertainty or hesitation rather than disagreement, as these respondents did not indicate negative perceptions of the telehealth process or program content.

Regarding acceptability, all participants (100%) agreed or strongly agreed that the suggested activities and home arrangements were appropriate and suitable for their infants and families. They emphasized that the therapist’s communication and approach toward both parents and infants were respectful and supportive, and that remote sessions were compatible with family dynamics and needs.

Within the satisfaction domain, 100% (N=14) of participants expressed that they were satisfied or highly satisfied with the program. They reported that the intervention effectively met their infants’ developmental needs and enhanced their confidence and competence as parents. All families stated that they would recommend the program to other parents.

Regarding safety, 100% (N=14) of families agreed or strongly agreed that all recommended activities were safe for their infants, that they received sufficient guidance to ensure a secure home environment, and that the therapist consistently attended to the infants’ and parents’ sense of safety. Additionally, 86% (n=12) indicated that no adverse incidents (such as falls or collisions) occurred during the program, while the remaining 14% (n=2) neither agreed nor disagreed, reporting no negative experiences but maintaining a neutral stance.

Qualitative comments from caregivers corroborated these quantitative results. Parents highly valued the program’s remote accessibility, the promptness of therapist replies, and the practicality of advice that could be incorporated into everyday routines. Initially, several families encountered difficulties integrating recommended activities into their routines and maintaining their infants’ attention during remote sessions; however, these obstacles diminished with time as they adapted to the approach. Participants characterized the program as coherent, efficient, and highly advantageous, noting that it not only facilitated their infants’ developmental progress but also enhanced their parental awareness, confidence, and involvement. Additionally, insights obtained from the 3 open-ended survey questions identified the following principal themes.

- The program’s most valued features: Parents consistently emphasized that the curriculum was lucid, pragmatic, and easily implementable within the home setting, using resources readily accessible in everyday life. They highlighted that the therapist’s instructions were clear and easily comprehensible, which enhanced their confidence in securely applying procedures. A mother remarked, “The activities were straightforward, and I was able to implement them immediately.” For instance, I discovered that my infant required visual exposure to his hands before using them, and that maintaining an upright posture was crucial for engaging with his surroundings. Another participant remarked, “I appreciated that I could utilize materials we already possessed at home and did not require any special purchases.” For example, I assisted my infant’s sitting with a laundry basket, used cardboard boxes as a diminutive table, and crafted vibrant rattles using lentils and plastic bottles—these were inventive, enjoyable, and secure concepts.” Numerous families appreciated the inclusive, home-centered format of the meetings, which facilitated the involvement of additional caregivers. One parent stated, “Due to its home-based nature, my husband and grandmother were able to participate, enhancing the experience and providing greater support for everyone involved.”
- Suggestions for program improvement: Although parents expressed predominantly favorable opinions, a minority proposed slight enhancements to improve involvement and continuity. They suggested creating a straightforward activity tracking checklist and a visual family guide featuring images and sequential examples of activities. These parents suggested that these tools would help them recall, organize, and confidently recreate activities between sessions. One participant stated, “A straightforward checklist would assist me in adhering to the activities more consistently and monitoring our weekly progress.” Another suggested, “A concise booklet featuring images that illustrate the proper positions and materials would enhance ease and safety in practice.” These recommendations indicated families’ need for organized assistance mechanisms to provide activities that enhance both safety and autonomy at home.
- Supplementary observations and pragmatic advantages: In their supplementary comments, parents underscored the practical and logistical benefits of the home-based arrangement. Avoiding traffic and congested public areas—particularly in major urban centers like Istanbul—was seen as alleviating stress, saving time, and reducing health risks for newborns. Families valued the flexibility to arrange lessons around their child’s daily schedule, including nap and feeding times. A mother remarked, “We no longer had to endure prolonged traffic, resulting in a calmer environment for both the baby and us, thereby enhancing the productivity of the sessions.” Another remarked, “The most advantageous aspect was the ability to select the most appropriate time for our child.” If the baby became fatigued, we could postpone the session to another time, which enhanced its effectiveness.

These thoughts emphasized that the program’s remote and home-based format not only improved safety and accessibility but also bolstered family engagement and comfort, strongly correlating with the favorable quantitative results.

### Young Children’s Participation and Environment Measure

Pre-post comparisons demonstrated statistically significant differences across both home and community participation domains. In the home setting, mean frequency scores increased from 3.67 (SD 0.37) to 4.32 (SD 0.62) (*P*<.001), and involvement scores increased from 2.43 (SD 0.57) to 3.49 (SD 0.47) (*P*<.001). Caregivers reported a decrease in desire for change (58.16, SD 5.63 to 21.43, SD 9.15; *P*<.001) and higher environmental support scores (78.75, SD 13.39 to 87.36, SD 7.35; *P*=.002).

In the community setting, frequency and involvement scores also showed statistically significant increases (*P*<.001), while desire for change decreased (58.91, SD 10.20 to 39.31, SD 10.30; *P*<.001). Environmental support scores increased from 68.13 (SD 12.75) to 76.19 (SD 13.95) (*P*<.001).

Overall, the YC-PEM captured measurable pre-post changes over the 12-week telehealth delivery period. Further details are presented in Table 2.

**Table 2:** Outcome changes after 12 weeks of intervention (N=14).

			BI^a^, mean (SD)		AI^b^, mean (SD)		Estimate of effect (95% CI)		*t* test (*df*)		*P* value
**YC-PEM^c^ Home**											
	Frequency	3.67 (0.37)		4.32 (0.62)		–0.65 (–0.96 to –0.35)		–4.655 (13)		<.001^d^	
	Involvement	2.43 (0.57)		3.49 (0.47)		–1.06 (–1.32 to –0.80)		–8.720 (13)		<.001^d^	
	Desire for change	58.16 (5.63)		21.43 (9.15)		36.74 (30.25 to 43.22)		12.243 (13)		<.001^d^	
	Environmental support	78.75 (13.39)		87.36 (7.35)		–8.61 (–13.31 to –3.91)		–3.954 (13)		.002^d^	
**YC-PEM Community**											
	Frequency	1.55 (0.51)		2.00 (0.54)		–0.45 (–0.64 to –0.26)		–5.036 (13)		<.001^d^	
	Involvement	1.46 (0.46)		2.29 (0.78)		–0.83 (–1.07 to –0.58)		–7.351 (13)		<.001^d^	
	Desire for change	58.91 (10.20)		39.31 (10.30)		19.60 (11.52 to 27.69)		5.236 (13)		<.001^d^	
	Environmental support	68.13 (12.75)		76.19 (13.95)		–8.06 (–11.41 to –4.71)		–5.195 (13)		<.001^d^	
**ASQ^e^**											
	Social emotional	32.50 (16.37)		16.79 (10.85)		15.71 (6.56 to 20.89)		2.393 (13)		<.001^d^	
	Communication	45.36 (8.65)		50.71 (6.75)		–5.36 (–10.22 to –2.38)		–2.252 (13)		.03^d^	
	Gross motor	32.50 (16.02)		48.21 (10.30)		–15.71 (–23.55 to –4.33)		–3.626 (13)		.001^d^	
	Fine motor	44.64 (11.00)		54.64 (4.98)		–10.00 (–16.98 to –3.09)		–3.231 (13)		.009^d^	
	Problem solving	46.79 (12.19)		53.21 (4.64)		–6.43 (–15.52 to 2.66)		–1.528 (13)		.15	
	Personal-social	38.93 (12.89)		51.79 (5.75)		–12.86 (–21.16 to –3.35)		–3.841 (13)		.005^d^	
AIMS^f^			31.07 (9.93)		47.50 (8.19)		–16.43 (–20.13 to –9.58)		–7.716 (13)		<.001^d^
**IMP^g^**											
	Variation	81.93 (6.81)		96.50 (4.80)		–14.57 (–19.04 to –10.10)		–7.041 (13)		<.001^d^	
	Adaptability	73.25 (8.95)		91.79 (9.40)		–18.08 (–23.25 to –12.92)		–7.706 (13)		<.001^d^	
	Symmetry	98.21 (5.21)		99.14 (2.18)		–0.93 (–2.93 to 1.08)		–1.000 (13)		.34	
	Fluency	88.71 (14.04)		99.14 (3.21)		–10.43 (–18.22 to –2.64)		–2.893 (13)		.01^d^	
	Performance	70.14 (9.35)		88.93 (6.60)		–18.79 (–22.29 to –11.59)		–11.587 (13)		<.001^d^	
	Total	82.86 (5.32)		95.00 (4.37)		–12.14 (–14.66 to –10.41)		–10.408 (13)		<.001^d^	
**DASS-21^h^**											
	Depression	3.29 (2.40)		1.64 (1.01)		1.64 (0.50 to 2.79)		3.097 (13)		.008^d^	
	Anxiety	2.36 (0.84)		0.57 (0.65)		1.79 (1.38 to 2.19)		9.555 (13)		<.001^d^	
	Stress	6.57 (2.68)		4.00 (2.22)		2.57 (1.67 to 3.47)		6.188 (13)		<.001^d^	
	Total	12.36 (4.80)		6.21 (3.31)		6.14 (4.64 to 7.64)		8.849 (13)		<.001^d^	

^a^BI: before intervention.

^b^AI: after intervention.

^c^YC-PEM: Young Children’s Participation and Environment Measure.

^d^Statistically significant results (*P*<.05).

^e^ASQ: Ages and Stages Questionnaire.

^f^AIMS. Alberta Infant Motor Scale.

^g^IMP: Infant Motor Profile.

^h^DASS-21: Depression Anxiety Stress Scales.

### Ages and Stages Questionnaire

Pre-post comparisons demonstrated statistically significant differences across several ASQ domains. Communication scores increased from 45.36 (SD 8.65) to 50.71 (SD 6.75) (*P*=.03), gross motor scores from 32.50 (SD 16.02) to 48.21 (SD 10.30) (*P*=.001), fine motor scores from 44.64 (SD 11.00) to 54.64 (SD 4.98) (*P*=.009), and personal-social scores from 38.93 (SD 12.89) to 51.79 (SD 5.75) (*P*=.005). Social-emotional scores decreased from 32.50 (SD 16.37) to 16.79 (SD 10.85) (*P*<.001).

Problem-solving scores increased over the 12-week period; however, this change did not reach statistical significance (*P*=.15). Overall, the ASQ detected measurable pre-post differences across multiple developmental domains during the telehealth delivery period. Further details are presented in Table 2.

### Alberta Infant Motor Scale

Pre-post analysis revealed a statistically significant difference in AIMS total scores over the study period. The mean total score increased from 31.07 (SD 9.93) at baseline to 47.50 (SD 8.19) at follow-up (*P*<.001). These results reflect measurable changes in gross motor performance across the 12-week telehealth interval.

### Infant Motor Profile

Pre-post comparisons indicated statistically significant differences across multiple IMP domains. Variation scores increased from 81.93 (SD 6.81) to 96.50 (SD 4.80) (*P*<.001), adaptability from 73.25 (SD 8.95) to 91.79 (SD 9.40) (*P*<.001), fluency from 88.71 (SD 14.04) to 99.14 (SD 3.21) (*P*=.01), and performance from 70.14 (SD 9.35) to 88.93 (SD 6.60) (*P*<.001). Symmetry scores did not differ significantly (*P*=.34). The overall IMP total score increased significantly from baseline to follow-up, from 82.86 (SD 5.32) to 95.00 (SD 4.37) (*P*<.001). Further details are presented in Table 2.

### Depression Anxiety Stress Scales

Analysis of caregiver-reported outcomes showed statistically significant differences between baseline and follow-up scores. Depression scores declined from 3.29 (SD 2.40) to 1.64 (SD 1.01) (*P*=.008), anxiety scores from 2.36 (SD 0.84) to 0.57 (SD 0.65) (*P*<.001), and stress scores from 6.57 (SD 2.68) to 4.00 (SD 2.22) (*P*<.001). The total DASS-21 score decreased from 12.36 (SD 4.80) to 6.21 (SD 3.31) (*P*<.001). Detailed results are presented in Table 2.

## Discussion

### Principal Findings

This study evaluated the feasibility, acceptability, satisfaction, and safety of delivering the HEP Approach through telehealth for premature infants at developmental risk. Fourteen of 16 enrolled infants completed the 12-week program (87.5% retention), and session adherence reached 100%, indicating strong feasibility. Between 86% and 100% of caregivers endorsed all feasibility, acceptability, satisfaction, and safety items. No adverse events were reported. Therapists likewise confirmed that the intervention was practical to deliver remotely and supported appropriate environmental adaptations. Qualitative feedback further emphasized the clarity of instructions, ease of implementation with household materials, logistical advantages (eg, avoiding travel and exposure to illness), and opportunities for broader family involvement. These findings are consistent with previous clinic-based feasibility research and randomized controlled trial outcomes examining the HEP Approach [24,40], supporting the model’s applicability across delivery formats.

The present results align with a growing body of telehealth-based early intervention literature demonstrating high parental engagement and satisfaction [31,33,34,57**-**59]. Telepractice models enabling routine-based home support have been well-received and promote active caregiver involvement [58]. Similar feasibility and satisfaction findings have been reported in the Baby Bridge telehealth model [32], neonatal intensive care unit follow-up telemedicine services [34], and telehealth physiotherapy programs for infants at risk of cerebral palsy [60,61]. Collectively, these studies reinforce the practicality and ecological validity of telehealth in early intervention contexts.

Significant improvements were observed in children’s participation, as measured by the YC-PEM. Increases in home and community frequency and involvement, alongside reduced caregiver desire for change and improved environmental support, suggest meaningful enhancement in functional engagement. These findings are consistent with prior studies demonstrating the YC-PEM’s sensitivity to intervention-related change across service models [62,63]. Similarly, previous HEP feasibility research using the Goal Attainment Scale reported functional improvements in daily routines [40]. Participation-focused enriched models such as the STEP protocol have also shown gains in YC-PEM outcomes [62,63], and early intervention intensity has been associated with improved home participation and environmental support [41]. Together, this evidence supports the relevance of participation-centered, family-focused intervention frameworks.

Motor development improved substantially. AIMS scores indicated significant gains in gross motor performance, consistent with previous clinic-based HEP studies [24,40]. These findings align with enriched environment-based interventions such as SAFE [21], STEP [62], and telehealth physiotherapy models [60], which have demonstrated positive effects on motor outcomes. The AIMS appears sensitive to motor change across both in-person and telehealth formats.

IMP findings further demonstrated significant improvements in variation, adaptability, fluency, performance, and total scores, reflecting enhancement in both motor quality and milestone acquisition. The absence of change in symmetry likely reflects high baseline symmetry, as similarly reported in CareToy studies [64]. Comparable improvements in IMP domains have been observed in enriched and family-centered models, including CareToy [64], COPCA [65], and EMI-Heart telehealth interventions [66]. Previous HEP studies have also demonstrated positive motor outcomes across multiple assessment tools [22,24,39,40,67]. Together, these results support the IMP’s responsiveness and reinforce the motor benefits of enriched, parent-guided early intervention.

ASQ findings revealed improvements in communication, gross motor, fine motor, personal-social, and social-emotional domains. These gains parallel performance-based motor improvements (AIMS and IMP) and are consistent with prior HEP research [24,40]. Similar multidomain improvements have been reported in enriched early intervention trials, such as the GAME [68,69] and structured physiotherapy programs [70], as well as in Alberta FICare models [71]. These findings suggest that family-centered enriched interventions may extend beyond motor domains, supporting broader sociocommunicative and cognitive development. In telehealth settings, the ASQ provides a practical, sensitive parent-reported measure for monitoring multidimensional progress.

Caregivers demonstrated significant reductions in depression, anxiety, and stress scores. These results mirror prior HEP findings [24] and align with evidence from other early intervention programs for parents of preterm infants [72]. The Mother-Infant Transaction Program [73], home-based postdischarge models [74], and family-centered neonatal care programs [75] have similarly reported improvements in parental psychological well-being. The consistent use of the DASS-21 across these studies supports its utility for detecting meaningful changes in caregiver emotional health.

Despite promising findings, several limitations must be acknowledged. The small sample size and lack of a control group limit causal inference and generalizability. Developmental maturation cannot be entirely excluded as a contributing factor. Long-term effects were not evaluated. Future studies should include larger randomized controlled trials, longitudinal follow-up, hybrid delivery models, cost-effectiveness analyses, and examination of cultural and socioeconomic influences on participation.

### Conclusions

This exploratory feasibility research established that the telehealth implementation of the HEP Approach is a feasible, safe, and well-received early intervention paradigm for premature infants at developmental risk. High retention, complete session adherence, and elevated caregiver satisfaction percentages demonstrate that remote implementation of the HEP Approach is both feasible and based on family needs. In addition to feasibility, notable enhancements were evident across various developmental domains, including gross and fine motor skills, communication, social-emotional development, participation outcomes, and caregiver psychological well-being. The findings indicate that the fundamental elements of the HEP model, which prioritize environmental enrichment, parental guidance, and active engagement, can be effectively adapted to telehealth formats without undermining therapeutic integrity.

Despite the promising results, they must be approached with caution due to the limited sample size and the single-group design. Future research must include larger randomized controlled trials and longitudinal follow-up to assess the long-term effects, cost-effectiveness, and generalizability of the telehealth-based HEP Approach across diverse target groups and cultural contexts. The current findings, however, offer initial evidence that the HEP telehealth model constitutes a promising and scalable approach to accessible, family-centered early developmental care for preterm infants.

## Data Availability

The data are not publicly available due to ethical and privacy restrictions, but are available from the corresponding author upon reasonable request.

## Appendix

Multimedia Appendix 1 Description of the Homeostasis–Enrichment–Plasticity Approach phases in telehealth delivery.

## Abbreviations

AIMS: Alberta Infant Motor Scale
ASQ: Ages and Stages Questionnaire
DASS-21: Depression Anxiety Stress Scales
EI: early intervention
HEP: Homeostasis–Enrichment–Plasticity
IMP: Infant Motor Profile
PEO: Person–Environment–Occupation
YC-PEM: Young Children’s Participation and Environment Measure
