# Supplementary material for: Telehealth Delivery of the Homeostasis–Enrichment–Plasticity Approach for Premature Infants With Developmental Risks: Exploratory Feasibility Study
Source: J Med Internet Res. 2026 Apr 7;28:e86883. doi: 10.2196/86883 (PMC13100579; doi:10.2196/86883)
Supplement: Multimedia Appendix 1 [file jmir_v28i1e86883_app1.docx]

**Table S1.** Description of the HEP® Approach phases in telehealth delivery.

| Program Phase | Description | Examples in Telehealth Context |
| --- | --- | --- |
| 1. Referral | Neonatologists or pediatricians refer infants meeting inclusion criteria. | Families were referred by neonatologists or pediatricians and subsequently contacted the research team for participation. Initial eligibility screening was conducted through telehealth interviews with caregivers, complemented by the review of digital versions of infants’ medical records. Eligible participants were infants born before 37 weeks of gestation, with a corrected age between 4 and 12 months, and without systemic disease or congenital disabilities. |
| 2.Family Introduction | Families were introduced to the philosophy of the HEP® Approach and the telehealth process through an initial online meeting, during which the family’s strengths and limitations were also discussed. | During the online meeting, clinicians introduced the HEP® Approach by explaining the program and providing families with a digital handbook. The discussion highlighted that every infant and family has unique strengths and limitations, and that building upon these strengths is critical to support the infant’s development. For example, when a baby demonstrates strong social skills, parents were encouraged to use these abilities to promote motor development. Similarly, families with larger households were guided to view this as an advantage, as it offers diverse opportunities to enrich the infant’s developmental experiences |
| 3.Comprehensive Assessment | Assessments were conducted remotely through structured caregiver interviews and guided observation of the infant via live video and parent-recorded clips. Standardized tests were administered online, and caregivers were asked to complete the relevant developmental scales digitally. | The assessment process was conducted via telehealth and included both spontaneous and structured components. Therapists first guided parents in positioning the camera appropriately to capture natural parent–infant interactions, observing the infant’s spontaneous movements, behaviors, preferences, and exploratory patterns, as well as parental responses. Parents were then instructed to place the infant in different positions and to present specific materials, enabling therapists to conduct structured assessments and standardized tests. In addition, developmental history was obtained through family interviews, which explored the infant’s past experiences, current services, future expectations, and both the physical and social environment. Family questionnaires were completed online together, and during these interviews the family’s social supports as well as their psychological and physical capacities were explored. |
| 4.Identification of Strengths and Challenges | Therapists collaboratively interpreted the assessment findings with parents via telehealth. The interpretation process aimed to identify underlying child, family, and environmental factors that might restrict the infant’s active exploration, while also recognizing the strengths that could be leveraged to support areas of vulnerability. This process sought to gain a holistic understanding of both the infant’s and the parents’ strengths and weaknesses across physical, psychological, social, and even financial domains, in order to determine which resources could be mobilized to promote the infant’s development and family well-being. | This stage emphasized identifying the infant’s and family’s strengths and limitations, and considering how these could be leveraged to support developmental progress. For instance, when social skills were recognized as a strength, parents were encouraged to use them to stimulate motor exploration in the presence of motor limitations. Likewise, if parents were highly motivated to engage but faced time constraints or physical limitations, existing family supports—such as an actively involved grandparent—were highlighted as valuable resources for fostering meaningful interactions. In this way, both infant and family strengths were reframed as opportunities to compensate for and address areas of vulnerability. |
| 5. Formulation of Hypotheses | In this phase, therapists formulated hypotheses based on assessment data to explain the underlying sensory–motor, emotional, cognitive, physical, and environmental factors contributing to the child’s strengths and limitations. These hypotheses were then shared with parents during telehealth sessions to clarify how such factors influenced the child’s development. | For example, one hypothesis suggested that the infant’s fear of falling was linked to heightened reactivity to movement, which limited attempts to change posture or explore new positions. Another hypothesis emphasized that insufficient waiting or attunement by caregivers—such as not maintaining eye level, speaking too quickly, or using limited gestures—contributed to restrictions in the infant’s social engagement. In addition, the infant’s limited manual exploration was hypothesized to stem from weak trunk control, as the infant primarily used their hands for postural support rather than for object exploration |
| 6.Collaborative Goal-Setting | Goals were set interactively during telehealth sessions using shared digital forms. Families were encouraged to articulate the changes they wished to see in their child, and examples were provided to illustrate how such priorities could be translated into specific developmental goals. This process emphasized identifying what was most meaningful for the family and collaboratively shaping these priorities into concrete, individualized goals. | Specific goals were collaboratively defined with families to address the infant’s developmental needs. For example, when weak trunk control limited manual exploration, a goal was set for the infant to increase independent use of the hands during sitting—from exploring objects only about 10% of the time to reaching 20–30%. Another goal focused on extending the infant’s sustained engagement with caregivers, aiming to increase interaction from 5 seconds to at least 10–15 seconds through strategies such as maintaining eye level, adjusting vocal tone, and using gestures. Additionally, for infants who avoided changing postures due to fear of falling, a goal was established for the child to attempt transitions between positions in at least two out of ten structured opportunities |
| 7.Identification of Outcome Measures | In determining outcome measures, families were actively engaged in a collaborative process to decide which tools would be used to evaluate their child’s progress. During telehealth sessions, clinicians explained the available developmental assessment instruments, and consensus was reached with parents on the most appropriate measures. This approach ensured that families understood how their child’s developmental gains would be tracked and fostered shared agreement on the evaluation process." | As an example of outcome measure sidentifcation, proximal developmental changes were evaluated with standardized tools such as the ASQ (Ages and Stages Questionnaire), AIMS (Alberta Infant Motor Scale), and IMP (Infant Motor Profile), which capture motor and broader developmental progress. Parental mental health and well-being were assessed with the DASS-21. Distal outcomes, reflecting functional participation and everyday engagement, were measured using the YC-PEM (Young Children’s Participation and Environment Measure) to evaluate the infant’s involvement in daily activities across home and community settings. |
| 8. Intervention Planning | During telehealth planning, clinicians collaborated with families to schedule weekly sessions at times that best suited the infant’s routines, the caregivers’ availability, and the therapist’s schedule—an essential advantage of the telehealth model. Session duration and expectations regarding parent-recorded videos and home practice were discussed in advance. Additionally, families and therapists jointly decided on the most appropriate physical setting for the sessions, including which room to use, the arrangement of the environment, and which caregivers (e.g., parents or grandparents) should be present to ensure meaningful participation. | For example, one family identified that the most suitable time for sessions was around noon on weekends, when the infant was most alert and multiple caregivers—including the grandmother as the primary daytime caregiver, along with the parents—could be present. They also agreed that the living room, where the infant spent most of their waking hours, was the most appropriate setting, ensuring both comfort and familiarity for the child during telehealth sessions. |
| 9. Intervention | Each telehealth session followed a structured sequence of phases. Sessions began with a brief check-in and feedback, where therapists and parents reflected on the past week, including observations from parent-recorded videos. In the second phase, parents were invited to freely interact with their infant, demonstrating natural routines without therapist direction, while ensuring appropriate camera placement for optimal observation. The third phase focused on guided reflection, where therapists highlighted the infant’s strengths and challenges and engaged parents through reflective questioning to explore their own interpretations. In the fourth phase, parents were encouraged to immediately apply insights gained from the discussion by practicing strategies in real time with their infant. Finally, sessions concluded with collaborative planning, setting expectations for home practice and identifying focal points for the upcoming session.  Within the telehealth format, session goals were structured progressively across the 12-week intervention. In the first 1–4 sessions, the primary focus was on supporting homeostasis and self-regulation by addressing basic needs such as sleep, feeding routines, a sense of safety, and regulatory capacity. Sessions 2–6 emphasized organizing both the physical and social environment of the home to facilitate active exploration, while also considering the unique characteristics of the infant and family. Between sessions 4–8, families were guided to diversify the infant’s emerging capacities by applying newly acquired skills in varied contexts, using different objects, and interacting with multiple caregivers. Finally, sessions 6–12 aimed to strengthen family self-efficacy by helping caregivers make appropriate environmental arrangements, adapt tools and objects, and adjust the level of task difficulty. Throughout the process, reflective questioning and real-time telehealth coaching supported parents in tailoring strategies to their infant’s developmental needs. | A sample telehealth session illustrates the phased structure of the intervention. In Phase 1 (check-in and feedback), the therapist welcomed the family, reviewed the previous week, and provided feedback from submitted videos—for example, noting that the infant sustained longer interactions, reached more actively, and showed signs of improved trunk control. In Phase 2 (free play observation), parents were invited to interact naturally with the infant while the therapist observed spontaneous play and exploration. In Phase 3 (reflective questioning), the therapist highlighted both strengths and subtle challenges, such as the infant’s hesitancy when releasing hand support, and encouraged parents to consider possible reasons—for instance, fear of falling. Parents were then guided to generate solutions, such as creating a safer environment with cushions or placing the infant in a secure container. In Phase 4 (parent practice), families applied these strategies in real time, testing their own ideas during the session. Finally, in Phase 5 (closure and planning), the therapist and family reflected on the outcomes, discussed the effectiveness of strategies, and outlined goals and activities for the following week |
| 10. Family Home Follow-Up and Monitoring | Families were supported in integrating the strategies into their daily routines at home and were asked to share at least one spontaneous video each week via digital platforms. These videos could capture moments of progress, ongoing challenges, or everyday interactions, without restrictions on context. Therapists reviewed the videos and provided individualized feedback, suggesting adaptations and encouraging families to generalize the strategies across different settings and situations. This process aimed to strengthen the infant’s learning and generalization skills while enhancing the family’s confidence in applying intervention strategies. | In one instance, the family spontaneously shared a video via WhatsApp in which the infant was observed actively exploring objects with their hands while seated in a small container. Although the infant demonstrated strong manual exploration, the video also revealed limited opportunities for postural changes and broader movement. Through follow-up conversations with the family on WhatsApp—both via messaging and phone calls—the therapist and caregivers jointly recognized that while the container provided security, it also restricted mobility. Together, they identified that introducing a larger container could preserve the infant’s sense of safety while enabling greater opportunities to explore varied movements and postures. |
| 11. Evaluation of Intervention Effectiveness | Post-intervention assessments conducted via telehealth, including re-administration of outcome measures and caregiver feasibility questionnaire. | At the final evaluation, families participated in an online interview that explored their experiences of the program, including perceived progress, remaining limitations, and overall reflections through open-ended questions. In addition, both unstructured and structured assessments were conducted via video: spontaneous interactions between the infant and caregivers were observed and documented, followed by standardized developmental tests. Parents also completed outcome measures and feasibility questionnaires, providing further evidence for the evaluation of the intervention. |
